# A New Handheld Device for the Detection of Falsified Medicines: Demonstration on Falsified Artemisinin-Based Therapies from the Field

**DOI:** 10.4269/ajtmh.16-0904

**Published:** 2017-05-03

**Authors:** Benjamin K. Wilson, Harparkash Kaur, Elizabeth Louise Allan, Anthony Lozama, David Bell

**Affiliations:** 1 Intellectual Ventures Laboratory, Bellevue, Washington; 2 London School of Hygiene and Tropical Medicine, London, United Kingdom

## Abstract

Poor-quality medicines are a major problem for health-care systems in resource-poor settings as identifying falsified medicines requires a complex laboratory infrastructure such as a Medicines Quality Control Laboratory. We report here an evaluation of a low-cost, handheld near-infrared spectrometer (NIRS) device by analyzing a library of artemisinin-based combination therapy (ACT) medicines to determine its usefulness as a drug-screening tool. The “SCiO” research prototype device was used to collect NIR spectra of a library of ACT and artesunate monotherapy medicine samples previously collected in Bioko Island and Equatorial Guinea and Kintampo, Ghana. The quality of these samples had been categorized as falsified, substandard, and quality assured based on the amount of stated active pharmaceutical ingredients detected using high-performance liquid chromatography photodiode array. Numerical analyses were performed on the NIR spectra to assess the usefulness of NIR to identify falsified and substandard medicines. The NIRS device was successful at detecting falsified medicines in all cases where the library contained both quality assured and falsified medicines of the same stated brand of medicines. The NIRS device was successful at identifying substandard amounts of artesunate but not amodiaquine in the ACT samples (*N* = 15) of artesunate–amodiaquine. This work reveals that this low-cost, portable NIRS device is promising for screening ACTs for falsified samples and could enable widespread drug screening at all points of the health system.

## Introduction

Drug quality is fundamental to the success of health interventions, with intentional or inadvertant provision of substandard and falsified medicines being a major contributor to delayed clinical recovery and mortality, particularly due to infectious disease in low-income countries.^1^ Use of medicines with subtherapeutic concentrations of the stated active pharmaceutical ingredient (SAPI) may drive microbial resistance. Although awareness of poor-quality medicines, in particular “fakes” has increased appreciably with the publication of reports citing that up to a third of antimalarials had failed chemical content analysis,^2^,^3^ the ability of health providers and consumers to detect substandard and falsified medication is limited by the high cost, limited accessibility, and destructive nature of testing.

Medicine quality is commonly divided into four classes: quality assured, falsified (also referred to as counterfeit), substandard, and degraded.^4^ Quality-assured medicines have the specified amount of SAPI and meet other quality attributes. Falsified medicines do not contain the SAPIs indicated on the packaging and generally do not comply with intellectual property rights.^5^ Substandard medicines are produced with poor constituents or manufacturing practice and may have contents or dissolution times that are outside of therapeutic limits,^6^,^7^ or have deteriorated due to adverse environmental conditions. Degraded formulations are commonly the result of exposure to light, heat, and/or humidity. It is clearly important to distinguish degraded from substandard products, as implications for rectifying these conditions are very different.^8^ In countries with insecure public or private supply chains, there is a need to detect at the point of importation as well as down the distribution chain to the point of purchase. Arguably, a place for consumer-level testing also exists in more regulated markets, where mail order medication (medicines bought over the internet) is becoming common practice.

Detecting the quality of medicines is a challenge for health-care professionals even though strategies and specifications have been formalized by international organizations such as the U.S. Pharmacopeia, the World Health Organization (WHO), and the Global Fund. Testing predominantly requires a well-equipped Medicines Quality Control Laboratory (MQCL) equipped with a range of analytical equipment such as high-performance liquid chromatography photodiode array (HPLC-PDA), mass spectrometry, dissolution testing apparatus, and so on.

Because of the cost and limited availability of MQCL, investigations are generally limited to relatively isolated and high-cost surveys and spot checking by large procurement agencies at or before the level of importation (missing the possibility of later substitution within the supply chain).^9^ Hence, there is a need for low-cost, robust, handheld devices for use as screening tools to detect poor-quality medicines with ease throughout the supply chain. Ideally, such a method would also be nondestructive. Devices fitting these criteria could transform pharmaceutical supply lines, providing assurance to health services, local providers, and consumers, while damaging the profitability of the drug counterfeit industry.^10^ Nondestructive spectroscopic techniques such as Raman spectroscopy and near-infrared spectroscopy (NIRS) are gaining interest for their ability to test drug samples without using the toxic chemicals or flammable solvents typically used in a MQCL, and with low training requirements.^11^ Raman and NIRS are limited to analyzing the state of the surface presented to the detector. Both techniques rely on comparing the characteristic spectral “fingerprint” of a suspect medicine with a quality-assured sample. To distinguish falsified from quality-assured medicines necessitates access to a database of spectra containing the spectral signature of the quality-assured product. NIRS, in particular, requires relatively complex computer analysis for library comparisons, as spectral features in this wavelength range are less pronounced than those observed by mid-infrared and Raman spectroscopy. Using NIRS to detect subtherapeutic levels of SAPIs necessitates that the specific chemical signature be distinguishable and quantifiable within the spectral pattern, and the surface presented be representative of the quantity within the whole product.

The TruScan^®^ by Thermo Fisher Scientific (Waltham, MA) has been investigated for drug-quality application in field settings and is equipped with software to identify certain artemisinin derivatives.^12^–^14^ However, adapting the TruScan as well as UV-Vis-NIR devices for the identification of falsified medicines in field settings has been limited.^15^ Portable Raman spectrometers are available for purchase for approximately USD 17,000–50,000, with costs driven by their relatively complex optics and necessity for a high-intensity illumination source.^11^ NIRS systems are simpler, requiring only a lamp for illumination and a dispersive optic, and are therefore more amenable to portability and low cost (approximately USD 1,000).

Herein, we evaluate the usefulness of a consumer NIRS technology presently marketed at USD 250 (“SCiO” research model device, Consumer physics, Israel), for its potential to detect poor-quality (substandard and falsified) first-line antimalarial medicines, specifically artemisinin-based combination therapies (ACTs). The ACTs used had been purchased in Bioko Island, Equatorial Guinea, and Kintampo, Ghana, and previously undergone quality testing.

## Materials and Methods

### The NIRS device.

**Figure 1.**
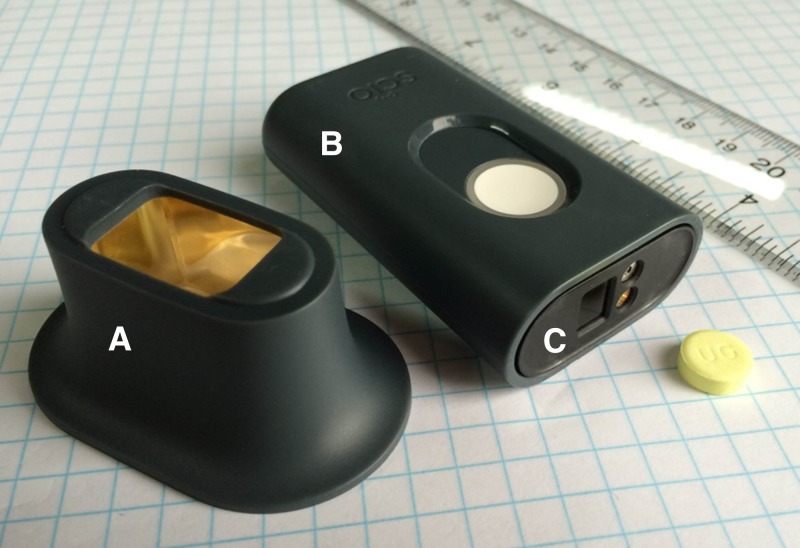
Photograph of the Consumer Physics near-infrared spectrometer (NIRS) research model. This image shows the (**A**) integrating attachment, (**B**) NIRS spectrometer, and (**C**) location of the light source and sensor on the NIRS. A dose of Lumartem^®^ (Cipla, India) is shown for scale.

The Consumer Physics SCiO research model NIRS device used in this study is a smartphone-sized instrument that promises to be highly effective at checking the quality of medications in the absence of a MQCL. It operates as a diffuse reflectance NIR spectrometer and includes a small, optical integrating attachment similar to an integrating sphere (Figure 1

). The device used in this work, a research prototype, was operated using a smartphone app.

### Field samples.

Artemisinin derivative-containing antimalarials, including ACTs and monotherapies, were purchased in Bioko Island, Equatorial Guinea. A total of 677 samples (77 brands) were purchased using three different systematic sampling approaches (convenience, mystery, and overt) from 278 outlets comprising of pharmacies, patent medicine vendors, public health facilities, and private hospitals (publication under review).

Samples were analyzed for content using the gold standard method of HPLC-PDA to quantify the amount of SAPIs present in each sample (SAPI 1 = artemisinin derivative, SAPI 2 = nonartemisinin antimalarial partner drug). Results were expressed as a percentage of the SAPI indicated on the packaging and samples were classified as quality assured, substandard, degraded, or falsified. The analysis showed that 7.4% of the samples were falsified, 1.6% were substandard, and 91.0% were quality assured. No degraded samples were detected (manuscript for publication in preparation).

Similarly, 257 artemisinin derivative-containing samples (31 brands) were purchased in Kintampo, Ghana, using the mystery client sample collecting approach. Analysis using the HPLC-PDA found 37.0% of the samples were substandard and 63.0% samples were quality assured. Quality-assured samples were so classified when they contained SAPIs within the tolerance band of 85–115% as measured by HPLC-PDA analysis and those out of this range were classified as substandard. The acceptable range for artemisinin-containing antimalarial (ACA) and its companion drugs according to the international pharmacopeia is for it to contain 90.0–110.0% SAPIs. However, the wider tolerance band was selected by the ACTc-DQ team to reflect the typically observed experimental variation in HPLC laboratory testing where less than 10 samples of each ACA formulation were tested.^7^ No falsified or degraded samples were detected.^16^

### NIRS spectrum acquisition.

Sample brands (Table 1) from Bioko Island, Equatorial Guinea, and Kintampo, Ghana, already analyzed for quality using the gold standard technique of HPLC-PDA were used to validate the usefulness of the NIRS device. The HPLC results were not shared at this stage with the persons using the NIR, ensuring that the study was blinded at this stage. Each ACA sample tablet under investigation (677 from Bioko Island and 257 from Kintampo, Ghana) was placed in the integrator attachment of the NIRS and scanned three times. Triplicate scanning reduces signal-to-noise ratio (by averaging scans together) and enables for the consistency of scan quality to be assessed. Bilayer tablets (all of which were Winthrop^®^ (Maphar, Morocco) artesunate–amodiaquine formulations) were scanned three times on each side and the two sides were considered independently for the analysis. The geometry of all the tested bilayer tablets was such that the two layers could be scanned separately using the integrating attachment.

Measurements were carried out in the 740- to 1,060-nm wavelength range, which is the full range of the NIRS device. The acquisition time for a single spectrum was around 2 seconds. The combination of the optical geometry of the integrating attachment and robust spectral preprocessing (described below) allowed spectra obtained with the integrating attachment to be consistent over a wide range of tablet positions within the chamber.

In addition, reference standard compounds of artemether, lumefantrine, artesunate, amodiaquine, dihydroartemisinin, and piperaquine were scanned without the integrating attachment (it is not possible to place them in the integrating attachment without contaminating of the interior surfaces).

### Spectral processing.

The goal of spectral processing is to remove extraneous signals such as background offsets and noise while enhancing spectral features. Averaged spectra were converted to absorbance units to enable linear fitting with reference standard compound in subsequent regression analysis. Spectra were then smoothed using a Savitzky–Golay filter, a common method that uses a sliding polynomial fit to reduce noise. For qualitative analysis, (identification) the second derivative spectra of absorbance were used to sharpen and enhance spectral features, a technique which produces improved performance in conjunction with library matching algorithms. For regression analysis of API concentration, the smoothed absorbance spectrum was used following a background subtraction.

### Analysis to identifying falsified samples.

The primary goal of qualitative analysis was to distinguish quality assured from falsified medicines. This was achieved using a library matching algorithm composed of a binary acceptance threshold based on Mahalanobis distance. Mahalanobis distance is a multidimensional distance measure that accounts for the mean and covariance of a target cluster (in this case a drug class). This is similar to the standard or *z*-score of a Gaussian distribution in one dimension and it can be used as a simple and robust measure of whether a sample under test belongs to a target cluster. Falsified medicines were excluded from all training sets, and cross-validation for testing quality-assured medicines was performed by excluding the batch under test from the training set. Excluding falsified medicines from the training set makes the resulting classifier more robust in real-world use, as a falsified drug could have any composition, not just those represented in the sample set.

### Analysis to identifying substandard samples.

The primary goal of regression analysis was to identify substandard medicines. Herein, three methods were used including a quantitative neural network, principal components regression, and partial least squares regression (PLSR). Of these three, PLSR showed the best results with the least tendency for overfitting. All quantitative results were obtained using leave-one-out cross-validation, or to predict the concentration of each sample a unique calibration curve was produced that did not include data from that sample.

## Results and Discussion

### Reference standard compounds versus ACTs.

**Figure 2.**
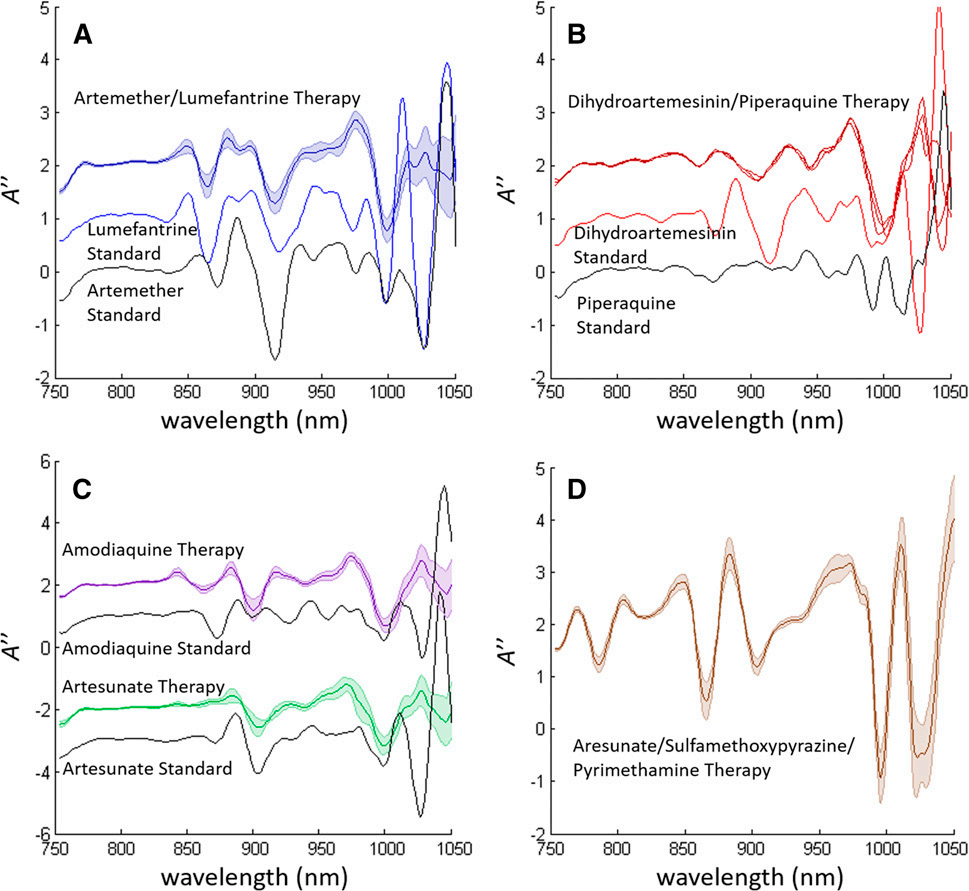
Second derivative absorption spectra of different active pharmaceutical ingredient (API) groups matched with the spectra of the associated API standards, when available. (A) Shows artemether-lumefantrine, (B) shows dihydroartemisinin-piperaquine, (C) show artesunate-amodiaquine, and (D) shows artesunate-sulfamethozypyrazine. Artesunate–amodiaquine is a combination therapy in which the two stated active pharmaceutical ingredients are formulated as either a bilayer or as separate tablets. This allowed their spectra to be collected and presented separately. The spectra in (C) represent scans from both bilayer tablet and multi-tablet doses.

NIR spectral analysis results of samples from Bioko Island were compared with those of the corresponding reference standard compound (Figure 2

). Note that formulations of artesunate–amodiaquine are shown as separate spectra despite being part of the same combination therapy, as these compounds (artesunate and amodiaquine salt) are unstable together, which necessitates for them to be formulated either as two separate tablets or as separate layers of a bilayer tablet.^17^,^18^ Figure 2C shows the combined spectra for both bilayer and multi-tablet therapies.

The spectral signatures shown in Figure 2A represent 12 brands of artemether–lumefantrine, whereas Figure 2C represents 16 brands containing artesunate and eight brands containing amodiaquine (see Table 1). The relatively narrow error bars represented in these plots indicate that the NIR signatures of drugs containing the same SAPI are consistent across different brands, whereas drugs containing different SAPIs have NIR signatures distinct from each other.

Spectral match of NIR peaks of the reference standard compounds and those from the samples of ACTs is shown for artemether–lumefantrine and artesunate (Figure 2A and C). However, this is not the case for peaks of amodiaquine, dihydroartemisinin, and piperaquine and tablets containing these medicines. This implies that a consistent formulation of excipients also contributes to the unique NIR spectral features associated with the SAPIs of the formulation as seen in Figure 2.

Quality-assured formulations of artesunate–sulfamethoxypyrazine–pyrimethamine combination therapy were also tested in the Bioko Island sample set to determine the representative spectrum (Figure 2D). Although there were no falsified samples or reference standards to show spectral comparisons, the pronounced spectral features of this formulation made it easy to identify versus all other formulations.

### Detection of falsified medicines.

**Figure 3.**
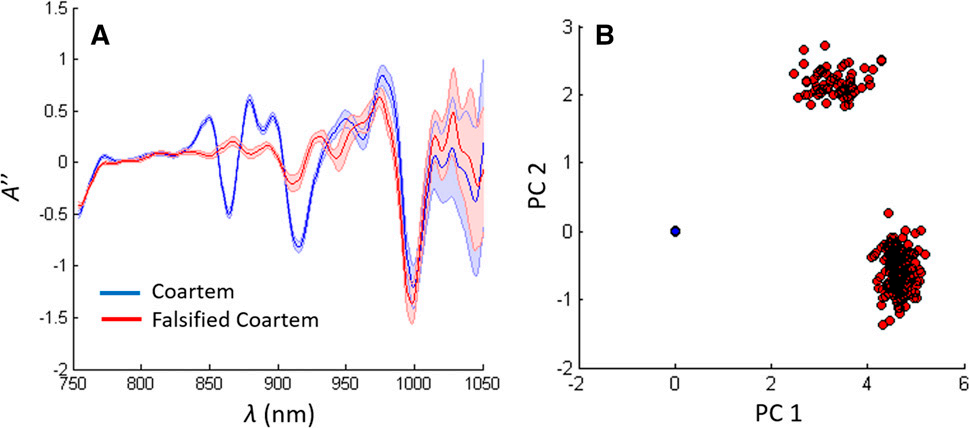
(**A**) Second derivative near-infrared spectra of quality assured and falsified Coartem. (**B**) Principal components clustering diagram for the spectra in (**A**).

The library matching algorithm described in the section analysis to identifying falsified samples was used to demonstrate the ability of a simple classifier to distinguish NIR spectra of quality-assured Coartem^®^ (Novartis, Cambridge, MA) from falsified Coartem samples RL (Figure 3

). The NIR spectrum of quality-assured Coartem is reproducible when compared with the falsified samples. This consistency in tablet spectra is most likely due to Coartem being produced by a manufacturer with WHO prequalification status. The Coartem cluster in Figure 3B (blue) contains 23 batches of quality-assured Coartem from two manufacturing locations (United States and China). Unsurprisingly, the library matching algorithm was able to distinguish quality assured from falsified Coartem with complete (100%) accuracy.

Artemisinin-based monotherapy tablets are not recommended for the treatment of malaria by WHO, but were purchased in Bioko Island. The library matching algorithm used was able to distinguish between the falsified and quality-assured monotherapy tablets (Table 2).

### Detection of substandard medicines.

**Figure 4.**
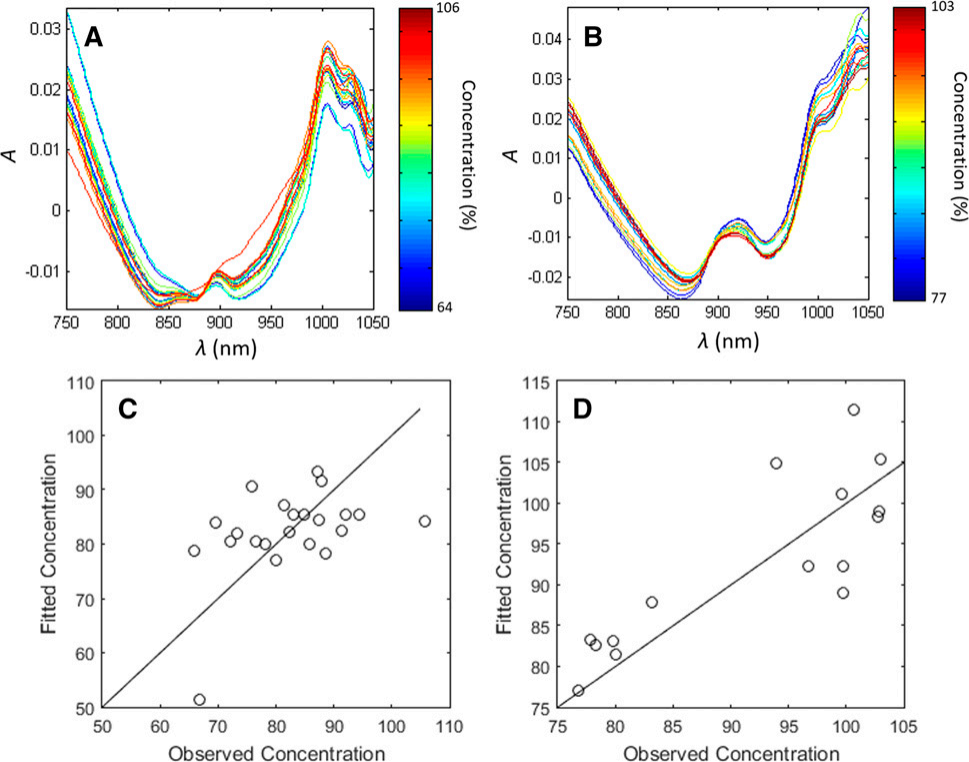
(**A**–**B**) Absorbance spectra of (**A**) amodiaquine tablets of Arsuamoon and (**B**) artesunate tablets of Combicure Adult. Concentration is indicated by color. (**C**) Results of partial least squares regression for amodiaquine in Arsuamoon and (**D**) artesunate in Combicure Adult. Concentrations are shown as a percentage of the labeled concentration. *R*^2^ values are 0.02 for (**C**) and 0.66 for (**D**). Fittings were based on three partial least squares modes.

Substandard medicines (i.e., low API concentrations), samples purchased in Kintampo, Ghana, that had been analyzed with HPLC-PDA and classified as substandard were used to assess the ability of NIRS to identify them (Table 3). Regression analysis of the NIRS spectra could not indicate the quantity of amodiaquine in the Arsuamoon^®^ [Guilin, China] brand (Figure 4

), as the spectra of the reference standard amodiaquine was not distinguishable in the spectral signature of therapies containing amodiaquine (Figure 2). In contrast, changes in the concentration of artesunate in the Combicure Adult brand (where artesunate and amodiaquine are formulated as separate tablets) could be detected, although the accuracy was low (*R*^2^ = 0.66). The Combicure Adult sample set had artesunate concentrations determined using HPLC-PDA ranging from 74.8% to 103% of SAPI, corresponding to doses of 74.8–103.0 mg. The quantitation accuracy of this linear model is defined by the standard deviation of the normalized residuals, which was 7.4%. This indicates that the NIRS can quantify the SAPI within ±14.8% with 95% certainty. However, the sample number was relatively low in this regression (*N* = 15) and a larger sample size is needed to determine the significance of this result. Figure 4A and B show the spectra of the two artesunate–amodiaquine brands, Combicure Adult and Arsumoon. Note that the spectra of Combicure Adult indicated systematic variation with SAPI concentration (shown by different colors), whereas the spectra of Arsuamoon does not.

## Conclusion

Falsified and substandard medicines are a major contributor to morbidity and mortality through treatment failure. The lack of a low-cost, simple, and reliable testing method to assess quality on the ground has restricted the ability of health and enforcement authorities to detect and deal with this issue. The results presented here indicate that handheld NIRS devices offer promise to address at least the first of these problems, that is, detecting falsified products provided that a consistent spectrum from the quality-assured product is available for comparison. NIRS also showed potential for detecting certain medicines with insufficient concentration of the SAPI and hence could be used for identifying substandard medicines, though we were only able to test the Combicure Adult and Arsumoon brands. These results require further validation and indicate that quantitation may not be possible for certain SAPIs, such as amodiaquine. Access to a wider NIR wavelength range of 740–1,060 nm will improve the detection of varying amounts of SAPIs. Handheld NIR spectrometers with wavelength ranges up to 2,400 nm are now available for sale.

The NIRS, coupled with the appropriate spectral library proved effective for identifying falsified samples of Coartem (artemether–lumefantrine) across multiple batches and manufacturing locations. Based on these results and the underlying technology, it seems likely that handheld NIRS scanners would be highly effective instruments for screening medicines from well-characterized brands with high-quality manufacturing (Coartem is prequalified by WHO). Falsified samples of other formulations or brands with matched quality-assured samples were only available for artesunate monotherapies.

The development of a library of spectra of quality-assured products approved by a national regulatory authority would allow low-cost rapid testing at port of entry and throughout the supply chain, as well as offer health providers and consumers with a means to confirm the authenticity of the product at point of use. Although technical developments in the near future are likely to enlarge this further, capacity to detect substitution of falsified products into supply chains should now be available to health systems and monitoring services with currently available technology.

## Figures and Tables

**Table 1:** Formulations of antimalarial medicines of varying brands and manufacturers tested using NIR spectroscopy

SAPI	Brand name	Manufacturer	Country
Artemether–lumefantrine	Alu	Ipca	India
	Artefan	Ajanta	India
	Artemef	Cipla	India
	Coartem	Novartis	China and United States
	Coatal	Jiangsu Yixing	China
	Cofantrine	Bliss GVS	India
	Colart	Ipca	India
	Combisunate	Ajanta	India
	Lonart-Ds	Bliss GVS	India
	Lufanter	Bliss GVS	India
	Lumartem/LumArt +	Cipla	India
	Tamether Fort	Jiangsu Yixing	China
Artesunate monotherapy	Actitesunate	Jiangsu	China
	Adamsnate	Adams	China
	Artesunat	Mekophar	Vietnam
	Artesunate 50	Aurochem	India
	Artesunate Tabs	Guilin	China
	Cusnat Artesunate	Green Energy	China
	Gricin	Greenfield	Chain
	Lever Artesunate	Adams	China
Artesunate–amodiaquine	Adamsnate Plus	Adams	China
	Arsuamoon	Guilin	China
	Art Amo Adult	Ipca	India
	ArtAm	Guilin	China
	Arthesis +	Cipla	India
	Asaq-Denk	Artesan	Germany
	Malatex	Kinapharma	Ghana
	Winthrop	Maphar	Morocco
Artesunate–sulfamethoxypyrazine–pyrimethamine	Co-Arinate	Famar Italia	Italy
	Malafan Plus	Kinapharma	Ghana
Dihydroartemisinin–piperaquine	Waipa Act	Kunimed	Nigeria

NIR = near-infrared; SAPI = stated active pharmaceutical ingredient.

**Table 2:** Falsified drug identification sample set and results

Brand name	SAPIs	Quality assured			Falsified		Sens/spec
		WHO PQ?	Samples	Country of manufacture (no. of batches)	Samples	Batches	
Coartem	Artemether Lumefantrine	Yes	60	United States (15) China (8)	22	3	100/100
Artesunat	Artesunate	No	3	Vietnam (1)	19	2	100/100
Cusnat	Artesunate	No	36	China (2)	1	1	100/100

SAPI = stated active pharmaceutical ingredients; sens/spec = sensitivity/specificity; WHO PQ = World Health Organization prequalified (manufacturer).

**Table 3:** Sample set for determining API concentration

Brand	API group	Substandard component	API range (%)	Samples	Batches	WHO PQ?
Arsuamoon	Artesunate–amodiaquine	Amodiaquine	66–85	15	7	Yes
Combicure Adult	Artesunate–amodiaquine	Artesunate	75–83	10	4	No

API = active pharmaceutical ingredient; WHO PQ = World Health Organization prequalified (manufacturer).
